# Outcome after ablation of atypical atrial flutter: Is induction a feasible approach?

**DOI:** 10.1016/j.ijcha.2024.101489

**Published:** 2024-08-14

**Authors:** N. Vonderlin, J. Siebermair, A.A. Mahabadi, D. Dobrev, T. Rassaf, R. Wakili, S. Kochhaeuser

**Affiliations:** a Department of Cardiology and Vascular Medicine, West German Heart and Vascular Center Essen, University of Essen Medical School, University Duisburg-Essen, Essen, Germany; b German Centre for Cardiovascular Research (DZHK), Germany; c Institute of Pharmacology, West German Heart and Vascular Center, University Duisburg-Essen, Germany; d Department of Molecular Physiology & Biophysics, Baylor College of Medicine, Houston, TX, United States; e Department of Medicine and Research Center, Montreal Heart Institute and Université de Montréal, Montréal, Quebec, Canada; f Department of Medicine and Cardiology, University Hospital Frankfurt, Goethe University, Frankfurt, Germany; g Department of Cardiology, Niels Stensen Kliniken, Marienhospital Osnabrück, Osnabrück, Germany

**Keywords:** Outcome, Induction, Atypical atrial flutter

## Abstract

**Background:**

Atypical atrial flutter (AAF) is an increasingly relevant clinical problem. Despite advancements in mapping and ablation techniques, the general management of these patients remain challenging especially when mapping cannot be performed during ongoing arrhythmia. There are no data whether induction of AAF is a feasible approach in these cases.

**Methods:**

We retrospectively analyzed patients who underwent catheter ablation of AAF and compared procedural results between patients with ongoing tachycardia when starting the procedure and patients with induced AAF.

**Results:**

We analyzed 97 ablation procedures performed in 76 patients with a mean follow-up of 13.2 ± 12.2 months. In 68 procedures (70.1 %) AAF was ongoing at the beginning of the procedure and in 29 cases (29.9 %) AAF had to be induced.

There was no statistically significant difference regarding acute procedural success. The recurrence rate of any arrhythmia during follow-up was significantly higher after ablation of ongoing AAF compared to induced AAF (63.2 % vs. 42.9 %; p = 0.047) driven by a significant higher rate of AAF-recurrence (57.4 % vs. 34.5 %; p = 0.039). The number of ablated tachycardias per patient as well as the number of de-novo tachycardias found during re-ablation showed no significant difference between both groups.

**Conclusion:**

Starting a procedure with ongoing arrhythmia did not result in better short- or mid-term outcome in patients undergoing AAF ablation. Furthermore, based on our results inducing AAF seems a legitimate approach for AAF ablation in patients presenting in sinus rhythm.

## 1 Introduction

Atypical atrial flutter (AAF) is a heterogenous group comprising atrial arrhythmias that can arise by different reasons. In many cases AAF is related to prior left or right atrial ablation procedures like pulmonary vein isolation (PVI). With increasing importance of ablation in the treatment of atrial arrhythmias the incidence of AAF is also growing constantly [5], [11]. After ablation of atrial fibrillation (AF) or cardiac surgery, incidence of AAF has been reported as high as 10 % [2], [3], [18]. Furthermore, atrial cardiomyopathy is a major contributor to fibrosis and scars and leads to the occurrence of atrial tachycardia (AT) [6].

Because the reentry of AAF can vary between individual patients significantly, it is important to determine the exact mechanism (e.g. macro- or micro-reentry) and location of every AAF. Different forms of AAF can even be present in one patient. Therefore, the ideal situation for the treatment of AAF seems to be performing electro-anatomical mapping of a sustained clinical tachycardia to determine the best therapeutic approach such as focal or linear ablation.

However, for several reasons it can be challenging to start a procedure with ongoing clinical tachycardia. AAF can terminate or degenerate into AF while preparing the procedure, acute cardioversion might be necessary because of hemodynamical instability, or a cath lab might not be available on short notice.

In clinical practice the question rises how patients should be managed that have documented AAF but are currently in sinus rhythm (SR) or even AF. Waiting for the next occurrence of the clinical AAF can be frustrating for patients and above-mentioned problems might be encountered several times until ablation can be realized. Alternatively, an electrophysiologic study can be performed to induce AAF through atrial stimulation. However, there is a risk that the induced tachycardia is not clinically relevant, resulting in unnecessary ablation, especially in patients with extensive arrhythmogenic substrate.

There is no data whether the short- and mid-term success is better when comparing ablations of patients with ongoing AAF and patients with induced AAF.

## 2 Methods

### 2.1 Study protocol

For this retrospective study, we analyzed patients undergoing ablation of AAF between April 2018 and January 2021 that were identified from our ablation database at the West German Heart and Vascular Center, Essen. All patients were followed up in our outpatient clinic as part of our clinical standard routine using 24 h-Holter-ECG and 12-lead-ECG or contacted by telephone to assess the occurrence of clinical recurrence of any arrhythmia. In case the documentation was not performed at our institution, relevant documents and ECGs were requested and reviewed.

This single-center cohort study was conducted at the University Hospital Essen, Germany, in accordance with the Declaration of Helsinki and its amendments and was approved by the institutional review board of the University of Essen (number 21-10341-BO). Written informed consent was obtained from all study participants.

### 2.2 Endpoint definition

The primary study endpoint was to evaluate the outcome of patients with induced AAF in comparison to patients with ongoing AAF when starting the procedure. Furthermore, we analyzed the type of recurrence during follow-up by 24 h-Holter-ECG or – if not available as a retrospective analysis – by 12-lead ECG. Moreover, the occurrence and results of repeat ablations at our institution was studied*.* We also evaluated if the recurrent AAF form was the same or de-novo compared to the AAF during previous procedure.

### 2.3 Electrophysiological study and mapping

If patients were treated with antiarrhythmic drugs, they were usually continued for at least 2–3 months after ablation and only discontinued in case of ongoing freedom of recurrence. An oral anticoagulation therapy was administrated at least >24 h before catheter ablation. Within less than 72 h before the procedure, atrial thrombus was excluded by transesophageal echocardiography in every patient. An activated clotting time of >300 s was aimed throughout the procedure.

In case of ongoing AAF, electro-anatomical mapping of the tachycardia was performed as described below. If AAF was not ongoing at the beginning of the procedure, AAF had to be induced from SR. Induction was achieved by programmed and/or burst stimulation from the coronary sinus. Additional application of orciprenaline was used at the decision of the investigator.

For the mapping of AAF we used the Orion™ multipolar basket catheter and the Rhythmia™ system (both Boston Scientific, Marlborough, MA). A local activation time (LAT) map was generated with automatic standard beat acceptance criteria based on the annotations-algorithm: (1) cycle length (CL) variation, (2) activation time difference variations between the coronary sinus (CS) electrograms (EGMs), (3) propagation reference (ΔR), (4) respiration, (5) QRS morphology “favorite beat” electrocardiogram (ECG), (6) mapping catheter movement, (7) electrogram stability compared to last beat, (8) tracking quality and (9) window. Entrainment maneuvers were performed when the underlying mechanism of the tachycardia was unclear and/or to identify bystanding circuits. To avoid termination or changes to the tachycardia repeated entrainment maneuvers to identify the critical isthmus were not routinely performed but only when deemed necessary by the treating physician.

Based on the activation map, the most likely dominant AAF mechanism and the location of the circuit were determined. In dependence of catheter stability, anatomical location and vulnerable structures located nearby, appropriate ablation sites were selected. In most cases, the selected ablation site was a narrow scope between scars and anatomical obstacles. Several ablation sites can lead to the termination of the AAF. The specific ablation site was chosen at the discretion of the operator. Ablation was performed with an irrigated RF-ablation catheter of the treating physician’s choice. Usually, ablations were performed using 45 W with the possibility of reduced energy settings at vulnerable locations.

The aim of ablation was an ablation line resulting in a bidirectional block independent of the selected isthmus as well as focal ablations if necessary [7], [8], [9]. Bidirectional block at every ablation line was checked by re-mapping with the multipolar mapping catheter in sinus rhythm or during pacing at appropriate locations. Furthermore, differential pacing was performed to assess bidirectional block.

### 2.4 Statistical analysis

All statistical analyses were performed using SPSS 28.0 (IBM Corporation, Armonk, New York, US). Continuous parametric variables are expressed means (±standard deviation) or median (1st Quartile, 3rd Quartile) in the case of non-normal distribution. Categorical variables are listed as number (percentage). For comparison-of-means, Pearsons Chi^2^ or Fishers-Exact-Test were used for categorical variables and the Mann-Whitney-*U* test for continuous variables. Distribution of normality in continuous variables was tested with the Shapiro-Wilks test.

P-values below 0.05 were considered statistically significant for all performed tests.

## 3 Results

### 3.1 Patient characteristics

For this study, 76 patients undergoing ablation were retrospectively identified from the ablation database. In total, our analysis included 97 procedures (mean 1.3 ± 0.6 per patient) during which a total number of 154 AAF-forms (mean 1.7 ± 0.9 per procedure) occurred. The mean follow-up time was 13.2 months (±12.2 months). Baseline characteristics for all procedures are listed in Table 1. Patients with ongoing AAF were – compared to patients with induced AAF – significantly younger, had a lower CHA_2_DS_2_-VASc-Score and were less likely to have a history of stroke, TIA or diabetes mellitus.

**Table 1:** Patient’s baseline characteristics for the complete cohort and stratified by ongoing AAF. PVI: pulmonary vein isolation; LVEF: left ventricular ejection fraction; AAD: antiarrhythmic drugs; TIA: transitory ischemic attack.

**Baseline characteristics per procedure**	**Overall n = 97**	**Ongoing n = 68**	**Induced n = 29**	**p**
Age (years)	71.2 ± 12.9	69.6 ± 13.1	75.2 ± 10.9	0.022
Female, n	52 (53.4 %)	35 (51.5 %)	17 (58.6 %)	0.52
Follow-up (months)	13.2 (±12.2)	14.2 (±13.2)	10.9 (±9.4)	0.59

*Previous cardiac procedures, n*				
PVI, n	59 (60.8 %)	42 (61.8 %)	17 (58.6 %)	0.77
Typical atrial flutter, n	40 (41.2%)	30 (44.1 %)	10 (34.5 %)	0.38
Atypical atrial flutter, n	29 (29.9 %)	25 (36.8 %)	4 (13.8 %)	0.029
Post-cardiovascular surgery, n	20 (20.6 %)	14 (20.6 %)	620.7 %)	0.96
LVEF, n	51.2 (±8.1)	51 (±8.5)	53.6 (±7)	0.83

*Medication (AAD), n*				
Beta blocker, n	90 (92.8 %)	63 (92.6 %)	27 (93.1 %)	0.94
Amiodaron, n	11 (11.3 %)	8 (11.8 %)	3 (10.3 %)	0.84
Flecainid, n	9 (9.3 %)	7 (10.3 %)	2 (6.9 %)	0.6
TIA/Stroke in history, n	15 (15.5 %)	7 (10.3 %)	8 (27.6 %)	0.031
Coronary artery disease, n	31 (32 %)	20 (29.4 %)	11 (37.9 %)	0.41
Arterial hypertension, n	82 (84.5 %)	57 (83.8 %)	25 (86.2 %)	0.77
Diabetes mellitus, n	13 (13.4 %)	5 (7.4 %)	8 (27.6 %)	0.007
CHA_2_DS_2_-VASc-Score, Median (IQR)	3 (3–5)	3 (2–4)	4 (3–5)	0.017
Implantable device, n	36 (37.1 %)	25 (36.8 %)	11 (37.9 %)	0.91

Most patients (83.5 %) had a previous cardiac procedure at the time of the ablation. In more than two third of cases (77.3 %), patients had another ablation before, which was mainly pulmonary vein isolation (PVI 60.8 %). A previous ablation of AAF was significantly more frequent among patients who entered the procedure with ongoing AAF (36.8 % vs. 13.8 %; p = 0.029).

Nearly all patients (92.8 %) were treated with a betablocker. Treatment with a specific antiarrhythmic drug was relatively scarce with amiodarone and flecainide used in 11.8 % and 7.9 % of cases, respectively.

### 3.2 Electrophysiological characteristics

**Fig. 1:**
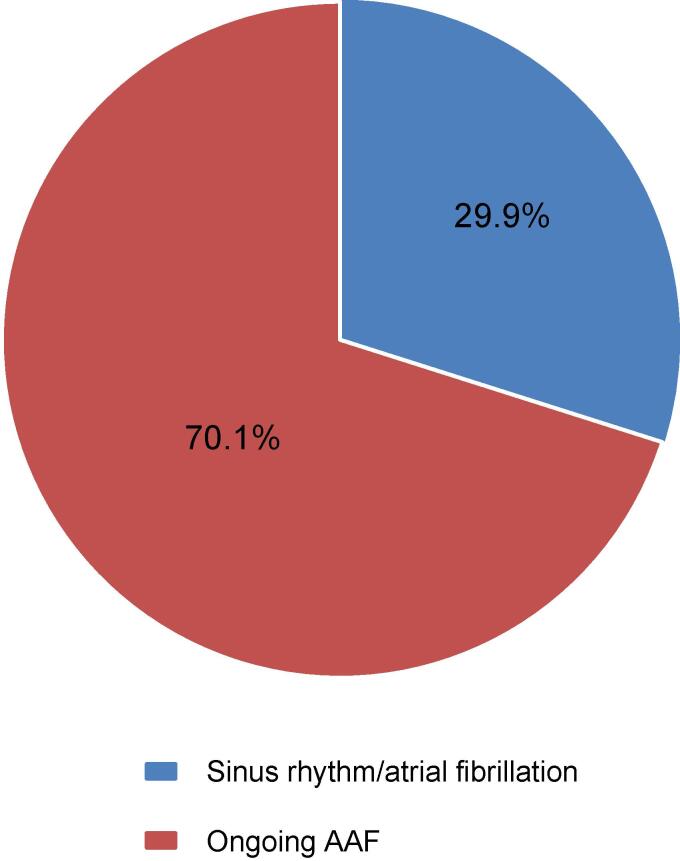
Rhythm when starting the procedure. The analysis includes 97 AAF forms. 68 AAF (70.1 %) were already ongoing when starting the procedure and 29 AAF (29.9 %) had to be induce. AAF: atypical atrial flutter.

The analysis included 97 procedures, in 68 cases with ongoing AAF being present at the beginning of the procedure and 29 procedures where AAF had to be induced (see Fig. 1). In nearly all cases (93.1 %), AAF was induced by programmed stimulation alone; an additional application of orciprenaline was only needed in one patient. In two patients an initial AF organized into a stable AAF that could be adequately mapped. Procedural characteristics are summarized in Table 2. Furthermore, Table 3 shows the different entities of AAF.

**Table 2:** Procedural characteristics for the complete cohort and stratified by ongoing AAF. AAF: atypical atrial flutter.

**Procedural characteristics**	**Overall n = 97**	**Ongoing** **n = 68**	**Induced** **n = 29**	**p**
Mean number of AAF/procedure, n	1.7 ± 0.9	1.7 ± 1.0	1.6 ± 0.7	0.87
Total fluoroscopic time, min	22.9 ± 12.6	21.3 ± 12.4	26.6 ± 12.6	0.85

Procedure result				
Sinus rhythm, n	96 (99.0 %)	67 (98.5 %)	29 (100 %)	0.51
Termination by ablation, n	86 (88.6 %)	60 (88.2 %)	26 (89.7 %)	0.84
Termination by cardioversion, n	9 (9.3 %)	6 (8.8 %)	3 (10.3 %)	0.81
Spontaneous termination, n	1 (1.0 %)	1 (1.5 %)	0 (0.0 %)	0.51
Redo-procedure	21 (21.6 %)	18 (26.5 %)	3 (10.3 %)	0.1
Same arrhythmia	12 (57.1 %)	11 (61.1 %)	1 (33.3 %)	0.55

**Table 3:** Entities of overall, ongoing and induced AAF: atypical atrial flutter.

**AAF-localisation**	**Overall** **n = 97**		**Ongoing** **n = 68**		**Induced** **n = 29**	
perimitral, n (%)	33	(34.0)	24	(35.3)	9	(31.0)
roof-dependant, n (%)	8	(8.2)	7	(10.3)	1	(3.4)
anterior scar, n (%)	10	(10,3)	3	(4.4)	7	(24.1)
posterior scar, n (%)	8	(8.2)	6	(8.8)	2	(6.9)
pulmonary veins, n (%)	27	(27.8)	19	(27.9)	8	(27.6)
bi-atrial, n (%)	1	(1.0)	1	(1.5)	0	(0.0)
right atrial, n (%)	9	(9.3)	7	(10.3)	2	(6.9)

In 96 of 97 cases the procedure resulted in a stable sinus rhythm. In most cases AAF was terminated by ablation (88.6 %), followed by cardioversion (9.3 %) while one AAF (1.0 %) terminated spontaneously. In one patient the procedure had to be aborted due to respiratory failure and did not result in SR. The patient recovered without consequences through conservative management. Acute bidirectional block was checked and achieved across all linear lesions as described above.

There were no statistically significant differences when comparing electrophysiological characteristics of ongoing AAF forms with induced AAF forms.

### 3.3 Recurrence rate of AAF

**Fig. 2:**
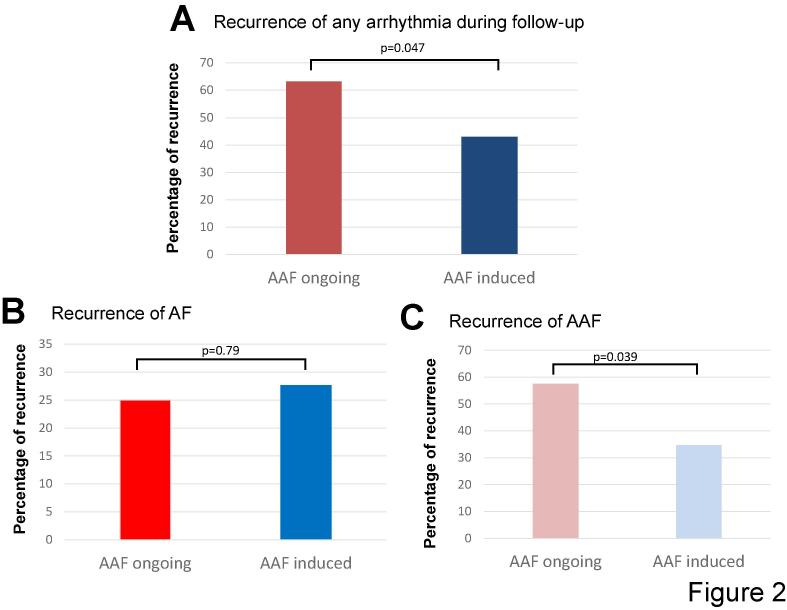
Recurrence rate of AT. (A) Shows the recurrence rate after ablation of ongoing AAF in comparison with induced AAF. There was a significant higher recurrence rate of any arrhythmia in patients with ongoing AAF (63.2 % vs. 42.9 %; p = 0.047). (B) The rate of AF recurrence showed no statistically significant difference (25.0 % vs. 27.6 %; p = 0.79). (C) Illustrates that the difference was driven by a higher rate of AAF-recurrence after ablation of ongoing AAF compared to ablation of induced AAF (57.4 % vs. 34.5 %; p = 0.039). AT: atrial tachycardia; AF: atrial fibrillation; AAF: atypical atrial flutter.

In a first step we evaluated the recurrence rate after ablation of ongoing AAF in comparison with induced AAF. During the follow-up, our analysis revealed a recurrence of any arrhythmia in 56.7 % of all cases. There was a significant higher recurrence rate of any arrhythmia in patients with ongoing AAF (63.2 % vs. 42.9 %; p = 0.047; see Fig. 2A). This difference was driven by a higher rate of AAF-recurrence after ablation of ongoing AAF (57.4 % vs. 34.5 %; p = 0.039). The rate of AF recurrence showed no statistically significant difference (Fig. 2B and 2C).

### 3.4 Patients with Re-Ablation in FU

Furthermore, we evaluated patients who underwent a re-ablation at our institution during follow-up. A redo-ablation was performed after 21 of 97 procedures (21.6 %). There was no significant higher rate of re-ablations in patients who were initially ablated with ongoing AAF compared to those with induced AAF (26.5 % vs. 10.3 %; p = 0.1).

**Fig. 3:**
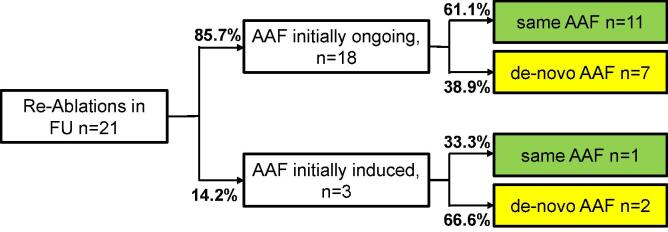
Patients with Re-Ablation in FU: A redo-ablation was performed in 21 of 97 procedures (21.6 %). 85.7 % of these re-dos were initially with ongoing AAF when starting the procedure and 14.3 % were with induced AAF in previous procedure. As shown, re-ablations after an initial ablation with ongoing AAF (n = 18) had in 61.1 % of cases the same AAF as in the previous procedure. In cases of induced AAF (n = 3), the rate of same AAF form was only 33.3 %. This difference was not statistically significant. FU: Follow-up; AAF: atypical atrial flutter.

We also compared whether the re-ablation showed the same arrhythmia as the previous ablation, or whether a de-novo tachycardia was found. Fig. 3 illustrates the distribution of the type of recurrent AAF and whether the initial ablation was started with ongoing AAF or induced AAF. Re-ablations after an initial ablation with ongoing AAF (n = 18) had in 61.1 % of cases the same AAF as in the previous procedure. In cases of induced AAF (n = 3), the rate of same AAF form was only 33.3 %. This difference was not statistically significant (Table 2).

## 4 Discussion

The management of patients with AAF is a special challenge. Next to the problems of a complex ablation procedure itself, the necessity to identify the individual mechanism of the tachycardia in every patient to allow targeted ablation approach remains a major issue. Lately, alternative approaches such as identification of deceleration zones during SR have emerged to predict sites that are crucial for tachycardia maintenance [17]. However, high density electro-anatomical activation-mapping of the running tachycardia, supported by entrainment maneuvers when appropriate, is still the gold standard to determine the specific mechanism and plan the best ablation approach [1], [10], [13]. Although several studies have investigated different approaches to the mapping and ablation of AAF [14], [15], so far there is no study that compared treatment success of AAF ablation in patients with ongoing AAF and induced AAF.

The acute success rate of AAF ablation in our study cohort was high and there was no significant difference between the two study groups. However, mid-term rates of AAF-recurrence were quite high, which is in slightly above the average of previous published results [4], [16]. A recently published study focusing on patients who had previously undergone catheter ablation or cardiac surgery reported that 54 % of patients experienced recurrence of either AAF or AFib [12]. The majority (83.5 %) of our study patients also had at least one previous cardiac procedure which means often a complex atrial cardiomyopathy with huge scar and a high number of co-morbidities.

Interestingly, the recurrence rate of AAF was significantly higher in patients with ongoing AAF when starting the procedure. Moreover, patients with ongoing AAF were significantly younger, had a lower rate of stroke and diabetes mellitus and a significantly lower CHA_2_DS_2_-VASc-Score. Although these findings suggest that the group of patients with ongoing AAF was younger and had less co-morbidities, it is still possible that the significantly higher recurrence rate in these patients is explained by electrophysiological characteristics not assessed in this retrospective analysis. Larger, prospective trials are needed to validate the finding of a reduced recurrence-rate after induction of AAF and elucidate possible explanations. Nevertheless, our data suggest that mid-term results of ongoing AAF is not necessarily superior to ablation of an induced tachycardia.

Inducing and ablating a tachycardia that is not clinically relevant during the treatment of AAF can be problematic for several reasons. Firstly, it leads to unnecessary ablation of tissue that is not responsible for the patient's symptomatic arrhythmia. This increases the risk of procedural complications. As a second point, ablation of non-critical areas can result in additional atrial scar promoting the development of new arrhythmogenic substrates. Consequently, this risk of future atrial arrhythmias increases and so aggravate the problem rather than resolving it.

In our group of patients, the number of treated arrhythmias per patient was not significantly different whether AAF was ongoing or induced during the procedure. When analyzing patients with re-ablation at our institution, we found a non-significant trend towards a higher number of redo-ablations after ablation of ongoing AAF. Interestingly, in about half of all redo-procedures the same form of AAF as in the previous procedure was diagnosed, while the other half consisted of de-novo tachycardias. Since bidirectional block was extensively checked at the end of all procedures, recovery of conduction over the applied ablation lesions seems to be a major cause for AAF-recurrence. However, some forms of atrial flutter, especially scar-dependent tachycardias can recur with very similar mechanism despite effective lesions. Additionally, the development of additional tachycardias, possibly due to extensive substrate is another relevant risk for recurrence. When comparing patients with an ongoing tachycardia during previous ablation and those who had to be induced, there was no significant difference in the amount of recurrence of the same tachycardia with a similar distribution between recurrent and de-novo AAF forms. Induction of AAF during the initial procedure seems not to result in a higher number of ablated AAF forms per patient or increased rate of recurrence of the initial tachycardia and seems to be a valid alternative approach for AAF ablation.

## 5 Limitations

We conducted a retrospective analysis from our ablation database. Therefore, we can only account for potential confounders that are available from our database. There might be an underestimation of recurrent rate as the FU was not standardized. Furthermore, we have no information about patients in which an induction of AAF was attempted but not successful and no ablation was performed. However, based on our experience this might not to be frequent, because in patients with a documented AAF a stable tachycardia can usually be induced.

Although the number of AAF is increasing with the number of left atrial ablation procedures, the overall number of patients presenting with AAF is comparatively low and the patient population is quite heterogenous.

Because the decision for the respective management approach was based on clinical considerations (e.g. hemodynamical stability during tachycardia) there might be significant selection bias. Patients with ongoing AFF might have a higher AAF burden compared to patients with induced AAF forms which could influence the recurrence rate.

Therefore, our study results do not allow extensive generalization but can serve as hypothesis generating for a prospectively randomized trial to validate our current findings.

## 6 Conclusion

The results of our study indicate that starting an ablation with an ongoing AAF is not associated with a better short- or mid-term rate of recurrence when compared to procedures with a tachycardia induced from SR. Furthermore, there was no indication that ablation of induced AAF resulted in an increased targeting of clinically irrelevant AAF forms. We therefore believe that this hypothesis generating study could warrant a larger, prospective study to validate the findings and further compare the different approaches to the management of AAF patients. A possibly approach might be the combination of induced AAF and prior identification of critical substrate during SR.

## Funding

This research did not receive any specific grant from funding agencies in the public, commercial, or not-for-profit sectors.

## Author contribution

Nadine Vonderlin and Simon Kochhaeuser: Involved in the conduct of the registry and in data acquisition, involved in data analysis and interpretation. Nadine Vonderlin, Johannes Siebermair, Amir Mahabadi, Dobromir Dobrev, Tienush Rassaf, Reza Wakili and Simon Kochhaeuser: Involved in critically revising the manuscript, have provided final approval, and take full accountability for the work, for all content and editorial decisions.

## 9 Study registration number

NCT06323499.

## CRediT authorship contribution statement

**N. Vonderlin:** Writing – original draft, Formal analysis, Data curation, Conceptualization. **J. Siebermair:** Writing – review & editing. **A.A. Mahabadi:** Writing – review & editing. **D. Dobrev:** Writing – review & editing. **T. Rassaf:** Writing – review & editing. **R. Wakili:** Writing – review & editing. **S. Kochhaeuser:** Writing – original draft, Formal analysis, Conceptualization.

## Declaration of competing interest

The authors declare that they have no known competing financial interests or personal relationships that could have appeared to influence the work reported in this paper.
